# Vaginal progesterone prophylaxis for preterm birth (the OPPTIMUM study): a multicentre, randomised, double-blind trial

**DOI:** 10.1016/S0140-6736(16)00350-0

**Published:** 2016-05-21

**Authors:** Jane Elizabeth Norman, Neil Marlow, Claudia-Martina Messow, Andrew Shennan, Phillip R Bennett, Steven Thornton, Stephen C Robson, Alex McConnachie, Stavros Petrou, Neil J Sebire, Tina Lavender, Sonia Whyte, John Norrie

**Affiliations:** a Tommy's Centre for Maternal and Fetal Health, MRC Centre for Maternal and Fetal Health, University of Edinburgh, Edinburgh, UK; b University College London, London, UK; c Robertson Centre for Biostatistics, Institute of Health and Wellbeing, University of Glasgow, Glasgow, UK; d Women's Health Academic Centre, King's College London, London, UK; e Imperial College London, London, UK; f Queen Mary University of London, London, UK; g Medical School, University of Newcastle, Newcastle, UK; h Division of Health Sciences, Warwick Medical School, University of Warwick, Coventry, UK; i University of Manchester School of Nursing, University of Manchester, Manchester, UK; j Centre for Healthcare Randomised Trials, Health Services Research Unit, University of Aberdeen, Aberdeen, UK

## Abstract

**Background:**

Progesterone administration has been shown to reduce the risk of preterm birth and neonatal morbidity in women at high risk, but there is uncertainty about longer term effects on the child.

**Methods:**

We did a double-blind, randomised, placebo-controlled trial of vaginal progesterone, 200 mg daily taken from 22–24 to 34 weeks of gestation, on pregnancy and infant outcomes in women at risk of preterm birth (because of previous spontaneous birth at ≤34 weeks and 0 days of gestation, or a cervical length ≤25 mm, or because of a positive fetal fibronectin test combined with other clinical risk factors for preterm birth [any one of a history in a previous pregnancy of preterm birth, second trimester loss, preterm premature fetal membrane rupture, or a history of a cervical procedure to treat abnormal smears]). The objective of the study was to determine whether vaginal progesterone prophylaxis given to reduce the risk of preterm birth affects neonatal and childhood outcomes. We defined three primary outcomes: fetal death or birth before 34 weeks and 0 days gestation (obstetric), a composite of death, brain injury, or bronchopulmonary dysplasia (neonatal), and a standardised cognitive score at 2 years of age (childhood), imputing values for deaths. Randomisation was done through a web portal, with participants, investigators, and others involved in giving the intervention, assessing outcomes, or analysing data masked to treatment allocation until the end of the study. Analysis was by intention to treat. This trial is registered at ISRCTN.com, number ISRCTN14568373.

**Findings:**

Between Feb 2, 2009, and April 12, 2013, we randomly assigned 1228 women to the placebo group (n=610) and the progesterone group (n=618). In the placebo group, data from 597, 587, and 439 women or babies were available for analysis of obstetric, neonatal, and childhood outcomes, respectively; in the progesterone group the corresponding numbers were 600, 589, and 430. After correction for multiple outcomes, progesterone had no significant effect on the primary obstetric outcome (odds ratio adjusted for multiple comparisons [OR] 0·86, 95% CI 0·61–1·22) or neonatal outcome (OR 0·62, 0·38–1·03), nor on the childhood outcome (cognitive score, progesterone group *vs* placebo group, 97·3 [SD 17·9] *vs* 97·7 [17·5]; difference in means −0·48, 95% CI −2·77 to 1·81). Maternal or child serious adverse events were reported in 70 (11%) of 610 patients in the placebo group and 59 (10%) of 616 patients in the progesterone group (p=0·27).

**Interpretation:**

Vaginal progesterone was not associated with reduced risk of preterm birth or composite neonatal adverse outcomes, and had no long-term benefit or harm on outcomes in children at 2 years of age.

**Funding:**

Efficacy and Mechanism Evaluation (EME) Programme, a Medical Research Council (MRC) and National Institute for Health Research (NIHR) partnership. The EME Programme is funded by the MRC and NIHR, with contributions from the Chief Scientist Office in Scotland and National Institute for Social Care and Research in Wales.

## Introduction

Several studies have assessed either vaginal progesterone or intramuscular 17α-hydroxyprogesterone caproate for the prevention of preterm birth in asymptomatic women with singleton pregnancy at high risk of preterm birth. An individual patient data meta-analysis of women with a short cervix showed that vaginal progesterone reduced the risk of preterm birth before 33 weeks (relative risk [RR] 0·58, 95% CI 0·42–0·80) and reduced a composite of neonatal mortality and morbidity (RR 0·57, 0·40–0·81).^1^ Although there is debate whether vaginal and intramuscular therapies have similar mechanisms or efficacy, the Cochrane Library meta-analysis groups the two treatments together, but reports separately for different maternal risk groups.^2^ Reduced risk of preterm birth before 34 weeks was shown in women with a short cervix (RR 0·64, 95% CI 0·45–0·90), without effect on perinatal mortality or neonatal death (perinatal mortality RR 0·74, 0·42–1·29; neonatal death RR 0·55, 0·26–1·13).^2^ By contrast, in women with previous preterm birth, progestogens reduced the incidence of preterm birth (RR 0·31, 95% CI 0·14–0·69), perinatal mortality and neonatal death.^2^ Although intramuscular 17α-hydroxyprogesterone caproate is licensed for women with a previous preterm birth, an independent analysis of data on vaginal progesterone for a US Food and Drug Administration advisory panel showed no benefit, with the panel concluding that “the overall risk/benefit profile [is] not acceptable” to support approval of vaginal progesterone in women with a short cervix.^3^

**Research in context**

**Evidence before this study**

Vaginal progesterone administration has been shown to reduce the risk of preterm birth and neonatal morbidity in women at high risk, but there is uncertainty about longer term effects on the child. We searched the Cochrane Pregnancy and Childbirth Group's Trials Register and the Cochrane Central Register of Controlled Trials (the Cochrane Library) until Feb 4, 2016, on MEDLINE (Jan 1, 1996, to Feb 4, 2016), and PubMed (Jan 1, 1974, to Feb 4, 2016) using the terms “progesterone/progestogen” AND “preterm birth prevention” AND “randomised trial” with no language restrictions. We also searched reference lists of trials and other review articles identified from this initial search and from our records. We excluded women with multiple pregnancy and those with symptoms of preterm labour. We identified two systematic reviews that compared preterm birth rates, neonatal outcomes, or childhood outcomes in women treated with progesterone or progestogens compared with those treated with placebo: a conventional meta-analysis published by the Cochrane collaboration and an individual patient data meta-analysis. No additional randomised trials were identified which were not included in the Cochrane review. Neither of the meta-analyses reported on our three primary outcomes, those of fetal death or delivery, either occurring before 34 weeks and 0 days of gestation (obstetric primary outcome); a composite of death, bronchopulmonary dysplasia, and brain injury on cerebral ultrasound (neonatal primary outcome); or the Bayley-III cognitive composite score at 22–26 months of chronological age (childhood primary outcome). One individual patient data meta-analysis of women with a short cervix reported the effect of vaginal progesterone on the outcomes of preterm birth before 33 weeks (relative risk [RR] 0·58, 95% CI 0·42–0·80), and on a composite of neonatal mortality and morbidity (RR 0·57, 0·40–0·81). This individual patient data meta-analysis was restricted to women treated with vaginal progesterone. The Cochrane Library meta-analysis grouped women treated with any progestogen and reported on risk of preterm birth before 34 weeks review for women with a short cervix (RR 0·64, 95% CI 0·45–0·90), and on perinatal mortality (RR 0·74, 0·42–1·29) or neonatal death (RR 0·55, 0·26–1·13). Regarding women with a previous preterm birth, the Cochrane Library reported that progestogens reduced the incidence of preterm birth (RR 0·31, 95% CI 0·14–0·69), and both perinatal mortality (RR 0·50, 0·33–0·75) and neonatal death (RR 0·45, 0·27–0·76). Neither the individual patient data meta-analysis nor the Cochrane review were able to report on childhood outcomes, with the Cochrane review noting that “there is limited information available relating to longer-term infant and childhood outcomes, the assessment of which remains a priority”.

**Added value of this study**

The OPPTIMUM study is, to our knowledge, the largest study to compare obstetric, neonatal, and childhood outcomes in high-risk women with singleton pregnancy treated with vaginal progesterone to prevent preterm birth. It is one of the few studies to look at childhood effects. In OPPTIMUM, by contrast with some of the published literature, vaginal progesterone was not significantly associated with reduced risk either of preterm birth or of composite neonatal adverse outcomes. Additionally, progesterone had no significant long-term benefit or harm on outcomes in children at 2 years of age. The primary outcomes reported in OPPTIMUM were different from the outcomes reported in the meta-analyses described above (and indeed different from the primary outcomes in the source studies), hence meta-analysis of the evidence to provide a meaningful pooled estimate was not possible. We plan an individual patient data level analysis that will be able to address complexities such as different inclusion criteria for the studies, different progestogens used (vaginal progesterone or 17α-hydroxyprogesterone caproate), and the differences in outcome reporting.

**Implications of all the available evidence**

The findings from OPPTIMUM are different to some of those reported in the literature. For the first time, we show childhood outcomes of progesterone to prevent preterm birth. The results of OPPTIMUM should prompt a major review of the use of progesterone for preterm birth prophylaxis, a search to identify specific women who might specifically benefit, and a redoubling of efforts to find alternative strategies to prevent preterm birth in women at risk.

Despite recommendations for progesterone use^4^ there are few data on long-term benefit or safety for the baby beyond the neonatal period. Adverse childhood effects of preterm birth include neurodevelopmental and cognitive impairments, and increase with degree of prematurity.^5^ Progesterone, by delaying birth and reducing prematurity, might reduce risk of impairment, but this could be offset by direct fetal harm by continuing prolonged exposure to intrauterine infection or inflammation, commonly associated with preterm labour. Furthermore, therapies applied in pregnancy might have differing effects in the neonatal period and early childhood (benefit in one and harm in another), as shown in the ORACLE II trial of antibiotics in spontaneous preterm labour^6^, ^7^ and in trials of multiple doses of corticosteroids.^8^ Hence, further information on childhood outcomes following progesterone treatment is required to determine the risk–benefit ratio of this therapy.

Therefore, we did a double-blind randomised trial to determine whether vaginal progesterone prophylaxis given to reduce the risk of preterm birth affects neonatal and childhood outcomes.

## Methods

### Study design and participants

OPPTIMUM (dOes Progesterone Prophylaxis To prevent preterm labour IMprove oUtcoMe?) is a multicentre randomised double-blind placebo-controlled trial. Women were recruited from 65 UK National Health Service hospitals and one Swedish hospital. An abbreviated protocol has been published.^9^

The study was granted approval by the Scotland A Research Ethics Committee (reference 08/MRE00/6). Clinical trials authorisation was given by the Medicines and Healthcare products Regulatory Agency (MHRA reference 22931/0009/001-0001 later revised to 01384/0208/001). A trial steering committee and a Data Monitoring Committee supervised the conduct of the study (appendix).

The study comprised a screening phase at 18–24 weeks and 0 days gestation and a treatment phase, starting at between 22 and 24 weeks of gestation. Written informed consent was obtained for both the screening phase (at 18–24 weeks and 0 days gestation) and treatment phase (between 22 and 24 weeks gestational age). All women had a singleton pregnancy, with gestational age established by ultrasound scan before 16 weeks, and were 16 years or older at screening. Women with clinical risk factors for preterm birth (any of a history in a previous pregnancy of preterm birth, or second trimester loss, or preterm premature fetal membrane rupture, or any history of a cervical procedure to treat abnormal smears) and a positive fetal fibronectin test at 22–24 weeks of gestation were eligible for random allocation in the treatment phase from the beginning of the trial, and designated fibronectin positive. After analysis of preliminary (masked) data in July, 2010, and the publication of a systematic review on screening for preterm birth,^10^ we realised that our initial selection strategy erroneously missed women at medium-to-high risk of preterm birth. Thus, from Sept 1, 2010, after recruitment of the initial 84 women, fibronectin-negative women with a history of spontaneous preterm birth at 34 weeks or less of gestation, or a cervical length of 25 mm or less were also eligible for inclusion, and designated a fibronectin-negative group (see appendix for detailed inclusion and exclusion criteria and fibronectin-positive or fibronectin-negative group allocation). There are no nationally agreed recommendations on which pregnant women should be screened for preterm birth risk by measuring cervical length, nor did the OPPTIMUM protocol include recommendations on who should undergo cervical length screening, hence any such measurements were made by clinicians on an individual patient basis before the woman's recruitment to OPPTIMUM. A cervical length of 25 mm or less at any time between 18 and 24 weeks and 0 days gestation in the index pregnancy conferred eligibility for recruitment.

### Randomisation and masking

Eligible women were allocated (1:1) to either progesterone 200 mg soft capsules (Utrogestan, Besins Healthcare) or an identical appearing placebo. Assignment to treatment allocation was done through a web portal hosted by the study data centre at the Robertson Centre for Biostatistics, at the Glasgow Clinical Trials Unit, University of Glasgow. The randomisation schedule was computer-generated at the Robertson Centre, using the method of randomised permuted blocks of length four, stratified by history of a previous pregnancy of more than 14 weeks of gestation and by study centre. Allocation concealment was achieved by use of a placebo, which appeared identical to the active drug. Participants were asked for informed consent and enrolled by collaborating clinicians (listed in this Article and the appendix), who used the web portal described above to randomly assign participants to treatment. Treatment allocation corresponded to a box number in the local pharmacy, containing either active or placebo drug. Participants, investigators, pharmacists, and others involved in giving the intervention, assessing outcomes, or analysing data remained masked to treatment allocation until the end of the study. There was no formal attempt made to assess the success of masking.

### Procedures

The participant administered the vaginal study medication daily at bedtime, commencing from about 22–24 weeks of gestation until 34 weeks or delivery of the baby, whichever was sooner. Co-administration of bromocriptine, rifamycin, ketoconazole, or ciclosporin was prohibited due to potential drug interactions. Rules for individual women to stop treatment on safety grounds (eg, after development of symptomatic placenta praevia) are defined in the protocol.

Compliance (assessed for each woman using a combination of medication pack returns, patient diaries, and patient self-reports) was calculated as the percentage of doses of study medication used divided by the expected doses. Adequate compliance was taken as 80% of prescribed medication.

Data were collected at screening, randomisation, 34 weeks of gestation, during labour and delivery, during the neonatal stay and at 1 and 2 years post-delivery to determine clinical outcomes. 2 year assessments, based on chronological age because of the mixed term and preterm population, were done at the local hospital clinic or at home. This assessment comprised the parent-completed structured clinical history, a parent-completed behavioural measure (the Strengths and Difficulties Questionnaire) and the cognitive scale of the Bayley Scales of Infant and Toddler Development 3rd Edition (Bayley-III). All assessments were undertaken by assessors who had received training, either from the study centre or via a national course; all met prespecified criteria of 90% agreement or more on an item-by-item basis with an independent psychologist. Record forms were checked centrally for consistency and completeness. For children for whom we could not arrange a clinic assessment we requested information from the family doctor concerning general health and the presence of motor, sensory, and developmental concerns.

### Outcomes

We defined three primary outcomes: either fetal death or delivery occurring before 34 weeks and 0 days of gestation (obstetric outcome); a composite of death, bronchopulmonary dysplasia, and brain injury on cerebral ultrasound (neonatal outcome); and the Bayley-III cognitive composite score at 22–26 months of chronological age (childhood outcome).

Brain injury was defined as any intraventricular haemorrhage (excluding subependymal haemorrhages), parenchymal cystic lesion or haemorrhagic lesion, or persistent ventriculomegaly (ventricular index >97th percentile). All scans were reported locally. All abnormal scans and 10% of normal scans were reviewed centrally masked to the local report (NM). Bronchopulmonary dysplasia (severe chronic lung disease) was defined as need for at least 30% oxygen or positive pressure (positive pressure ventilation or nasal continuous positive airway pressure) at 36 weeks postmenstrual age or discharge, whichever came first.

Secondary efficacy and safety outcomes were as follows: gestational age at delivery (weeks); deaths up to 2 years of age; death after trial entry up to the end of study; daily category of care after delivery room (normal or special or high dependency or intensive); surfactant administration; suspected or confirmed necrotising entercolitis; neonatal infections (one or more discrete episodes with positive blood culture among those with infection, one or more discrete episodes with positive CNS culture among those with infection); maternal or child serious adverse events during pregnancy and birth; composite outcome of death or moderate-to-severe neurodevelopmental impairment at 2 years; moderate-to-severe neurodevelopmental impairment; individual components of disability; admissions to hospital during follow-up; behavioural scale scores at 2 years assessed in strengths and difficulties questionnaire; change in EuroQol 5D (EQ-5D) from baseline to birth; change in EQ-5D from baseline to 12 months; and women's perception of treatment 1 month post-delivery (the proportion extremely or fairly satisfied). Outcomes were categorised as moderate or severe using published definitions.^11^

### Statistical analysis

A statistical analysis plan was finalised before data lock. Statistical analyses were done by C-MM and AM at the Robertson Centre for Biostatistics, Glasgow University according to the intention-to-treat principle. The three primary outcomes and secondary outcomes were compared between the treatment groups using mixed effects logistic regression (or, for continuous variables, linear regression) models including treatment allocation and previous pregnancy (≥14 weeks) as fixed effects, with study centre as a random effect. According to the prespecified statistical analysis plan, p values were initially reported without adjustment for multiple comparisons, then adjusted using a Bonferroni-Holm procedure.^12^ The planned sample size was around 1125 participants, depending on the relative numbers of fetal fibronectin-positive and fetal fibronectin-negative women recruited.^9^ Detailed sample size calculations are available in the published protocol, but in brief the study had at least 80% power to detect what was considered the minimal important clinical difference for each of the three primary outcomes at a nominal 5% level of significance.^9^

Sensitivity analyses included repeating the primary analyses in a per-protocol dataset (which excluded data from women who were found not to be compliant with the inclusion or exclusion criteria, or who had a structural or chromosomal fetal anomaly discovered after inclusion, or who had a multiple pregnancy discovered after inclusion or who were not adequately compliant with treatment by the prespecified definition), and the use of multiple imputation of missing primary outcome data. Preplanned subgroup analyses for primary outcomes were done by extending the main regression models to include interaction terms for the following subgroups: fibronectin positive or fibronectin negative, cervical length of at most 25 mm or longer than 25 mm, cervical length of at most 15 mm or longer than 15 mm, chorioamnionitis yes or no, history of spontaneous preterm birth or no such history, and history of preterm birth or no such history. Safety outcomes (adverse events) were assessed in a safety population, excluding women for whom it was documented that no study medication was taken. This trial is registered with ISRCTN.com, number ISRCTN14568373.

### Role of the funding source

Neither the funders of the study nor the provider of active and placebo medication had any role in study design, data collection, data analysis, data interpretation, or writing of the report. C-MM and AM had full access to all the data in the study and JEN had final responsibility for the decision to submit for publication.

## Results

We reviewed the case notes of 15 132 women for eligibility, between Feb 2, 2009, and April 12, 2013. 1228 (8%) were subsequently randomly assigned, 610 allocated to placebo and 618 to progesterone (figure). Two of these women were randomised in error and were excluded from initiating on treatment and the intention-to-treat population. Baseline characteristics of participants in the intention-to-treat population were balanced across the two allocated groups (table 1). The number of women randomly assigned per site ranged from one to 165; three sites screened but did not randomly assign participants. Information on the obstetric, neonatal, and childhood primary outcomes was available for 1197 (97%), 1176 (96%), and 869 (71%) of participants, respectively. There were few differences in baseline characteristics between those for whom primary outcome data was or was not available (appendix).

Information from diary returns for 1011 (82%) women showed 80% or more compliance in 361 (71%) of 509 in the placebo group and 333 (66%) of 502 in the progesterone group. For compliant women, the median percentage of medication taken was 92·3% (IQR 71·6–98·7) and 92·9% (59·0–98·6), respectively. No woman terminated treatment because of prespecified discontinuation rules.

Although the point estimate of the odds ratio (OR) was in the direction of benefit, administration of progesterone did not significantly alter the risk of the obstetric (fetal death or birth before 34 weeks) or neonatal (a composite of death, brain injury, or bronchopulmonary dysplasia) outcome after the prespecified adjustment for multiple comparisons (Bonferroni-Holm procedure): OR 0·86 (95% CI 0·61–1·22) for the obstetric outcome and OR 0·62 (0·38–1·03) for the neonatal outcome (table 2). Similarly, there was no effect on childhood outcomes (cognitive score 97·7 [SD 17·5] for placebo and 97·3 [17·9] for progesterone; difference in means −0·48, adjusted 95% CI −2·77 to 1·81).

Among the components of the primary obstetric and neonatal outcomes, the proportion of babies with observed neonatal brain injuries on cerebral ultrasound scanning was lower in the progesterone group (18 [3%] of 584 *vs* 34 [6%] of 574; OR 0·50, 95% CI 0·31–0·84; table 2). A reduction in brain injury was also observed in a sensitivity analysis restricted to participants in whom a neonatal brain scan was done (n=776; OR 0·54, 95% CI 0·32–0·88). Neonatal death was also less common in the progesterone group, but the low numbers precluded planned adjustment for the covariate previous pregnancy at 14 weeks or longer gestation.

Similar results for primary outcomes were achieved in per-protocol analyses (687 [56%] of 1226 patients in the intention-to-treat population; appendix); in analyses with multiple imputations of missing data on the primary outcomes (appendix); and in alternative multiple comparison procedures, including the Sidak-Holm method and permutation adjustment (50 000 permutations; data not shown). Comparison of characteristics of women included and not included in the per-protocol analysis are shown in the appendix. An additional sensitivity analysis with imputations for the variable smoking was done post hoc because of the difference in smoking prevalence in those with and without outcome data: again this generated similar results to the main analysis (data not shown). A post-hoc survival curve of time to death or delivery (primary obstetric outcome) showed that the differences between the progesterone and placebo groups appeared greatest at our prespecified gestational cutoff of 34 weeks (appendix).

Rates of preterm birth were higher in the predefined subgroups of women with a positive fetal fibronectin test, women with a cervical length of at most 25 mm, and women with a cervical length of at most 15 mm (appendix). However, there were no significant interactions between these groups and the effect of progesterone on any of the obstetric, neonatal, or childhood outcomes. Within subgroups there was no significant effect of progesterone on any of the primary obstetric or childhood outcomes (table 3). The interaction term approached statistical significance (p=0·053) for the neonatal outcome in the subgroup with a history of a previous spontaneous preterm birth, in which the OR for the neonatal outcome was lower in the progesterone group (0·48, 95% CI 0·29–0·79) compared with the complementary group with no previous spontaneous preterm birth (1·22, 0·55–2·71). However, caution is needed in interpreting all these findings given the number of prespecified subgroup analyses undertaken on three primary outcomes.

Most of the other secondary outcomes did not differ statistically between progesterone and placebo groups (table 4). Although neurodevelopmental impairments were similarly distributed in each group, somatic impairments in renal, gastrointestinal, and respiratory systems though of low frequency, were more common in the progesterone group. There were no apparent differences in the proportions with safety or other outcomes between the placebo and progesterone groups (table 5).

## Discussion

OPPTIMUM is the largest randomised trial of vaginal progesterone for prevention of preterm birth in women at high risk. By contrast with published reports,^13^, ^14^, ^15^ we show no effect of progesterone on rates of either preterm birth or neonatal composite outcome. For the first time using a direct assessment, we provide strong evidence that the use of progesterone from 22–24 to 34 gestational weeks has no demonstrable effect on 2 year neurodevelopmental outcomes, either as cognitive scores or impairments, suggesting that progesterone prophylaxis to prevent preterm birth appears safe for the baby (at least up to 2 years of age). Only one previous study has determined long-term effects of progestogens given to singleton pregnancies in a randomised trial of intramuscular 17α-hydroxyprogesterone caproate,^16^, ^17^ but this study used parent report and had a smaller sample size with a higher proportion lost to follow-up. The other published studies are restricted to questionnaire or health record-based assessments in twins whose mothers were enrolled in randomised trials of progesterone versus placebo.^18^, ^19^

OPPTIMUM was a pragmatic trial, set up to examine effects of progesterone on outcomes in a heterogeneous group of women at risk of preterm birth. We extended our recruitment criteria early in the study, when newly available information suggested we were missing women at high risk of preterm birth. Notably, the fibronectin-negative group recruited under the extended criteria, had rates of the primary outcome (death or preterm birth before 34 weeks) of 13% (appendix), some three times those of the background population of pregnant women in the UK.^20^ Hence, our decision to extend the recruitment criteria appears correct. Importantly, although we were able to define at baseline subgroups of women with higher rates of preterm birth (including those with a short cervix and those with a positive fibronectin test), our data suggest that the efficacy of progesterone (for all outcomes) is similar across groups. Therefore, our data do not support the premise that vaginal progesterone is specifically effective in women with a short cervix.

Although we showed no overall effect, point estimates of the reduction in the odds of the obstetric outcome (0·86) and the neonatal composite outcome (0·62) are in the direction of benefit, but with CIs that show no advantage. Additionally, point estimates in the short cervix subgroups are similar to those reported in meta-analyses of the effect of progesterone in such women. For example, the OR for preterm birth prevention was 0·69 in OPPTIMUM, compared with a RR of 0·64 (before 34 weeks) in one systematic review ^21^ and a RR of 0·58 (before 33 weeks) in an individual patient data meta-analysis.^1^ The corresponding figures for effects on a neonatal composite are OR 0·54 in OPPTIMUM and RR 0·57 in the individual patient data meta-analysis.^1^ An individual patient data meta-analysis, including the OPPTIMUM findings, to understand what the totality of evidence indicates, particularly within subgroups of interest, is likely to be helpful.

Although we have shown no significant effect on the overall neonatal composite outcome, there appeared to be a reduction in neonatal brain injury. Progesterone-associated reduction in brain injury is plausible given supportive preclinical data in adult models showing potentially neuroprotective effects of reduced inflammatory cytokine production, reduced activation of microglial cells, and limited apoptosis,^22^, ^23^ although a recent trial of over 1000 adult participants with traumatic brain injury has shown no clinical therapeutic effect.^24^ However, in the absence of long-term improvements in cognitive function, a protective effect of progesterone on brain injury (defined by ultrasound) might not be important clinically: not only was brain injury on ultrasound a relatively rare event in OPPTIMUM but other studies have shown no correlation between this finding and longer term neurosensory impairment.^25^ Additionally, these non-significant reductions in the neonatal composite adverse outcome need to be considered against the non-significant increase in the childhood adverse outcome of death or moderate-to-severe neurodevelopmental impairment.

OPPTIMUM strongly suggests that the efficacy of progesterone in improving outcomes is either non-existent or weak. Given the heterogeneity of the preterm labour syndrome we cannot exclude benefit in specific phenotypic or genotypic subgroups of women at risk. However, the subgroups of women who might benefit do not appear to be easily identifiable by current selection strategies, including cervical length measurement and fibronectin testing.

Reassuringly, our study suggests that progesterone is safe for those who wish to take it for preterm birth prophylaxis. The overall rate of maternal or child adverse events was similar in the progesterone and placebo groups. There were few differences in the incidence of adverse secondary outcomes in the two groups, with the exception of a higher rate of renal, gastrointestinal, and respiratory complications in childhood in the progesterone groups. Importantly, the absolute rates of these complications was low. Follow-up of other babies exposed in utero to vaginal progesterone would be helpful in determining whether the increased rate of some renal, gastrointestinal, and respiratory complications is a real effect or a type I error.

A potential weakness in our trial is that overall compliance was only 69%. This contrasts with a reported compliance of 88·5% in the study by Hassan and colleagues,^13^ but is greater than the compliance seen in routine clinical practice.^26^ Additionally, the assumption in the Hassan study that women who did not return study medication were fully compliant might have erroneously inflated their estimate of compliance. No information on compliance was reported in the other large study on vaginal progesterone in singletons.^15^ Notably, in OPPTIMUM, the effect size for each of the primary outcomes was very similar in the per-protocol analysis (restricted to those with adequate treatment compliance) compared with the intention-to-treat group, suggesting that suboptimum compliance did not have a major effect on overall results.

We believe that OPPTIMUM should prompt a major review of the use of progesterone for preterm birth prophylaxis, a search to identify specific women who might specifically benefit, and a redoubling of efforts to find alternative strategies to prevent preterm birth in women at risk. For those clinicians and women who wish still to use progesterone for preterm birth prophylaxis, our data provide reassurance that it appears safe, at least until 2 years of age of the child.

## Figures and Tables

**Figure:**
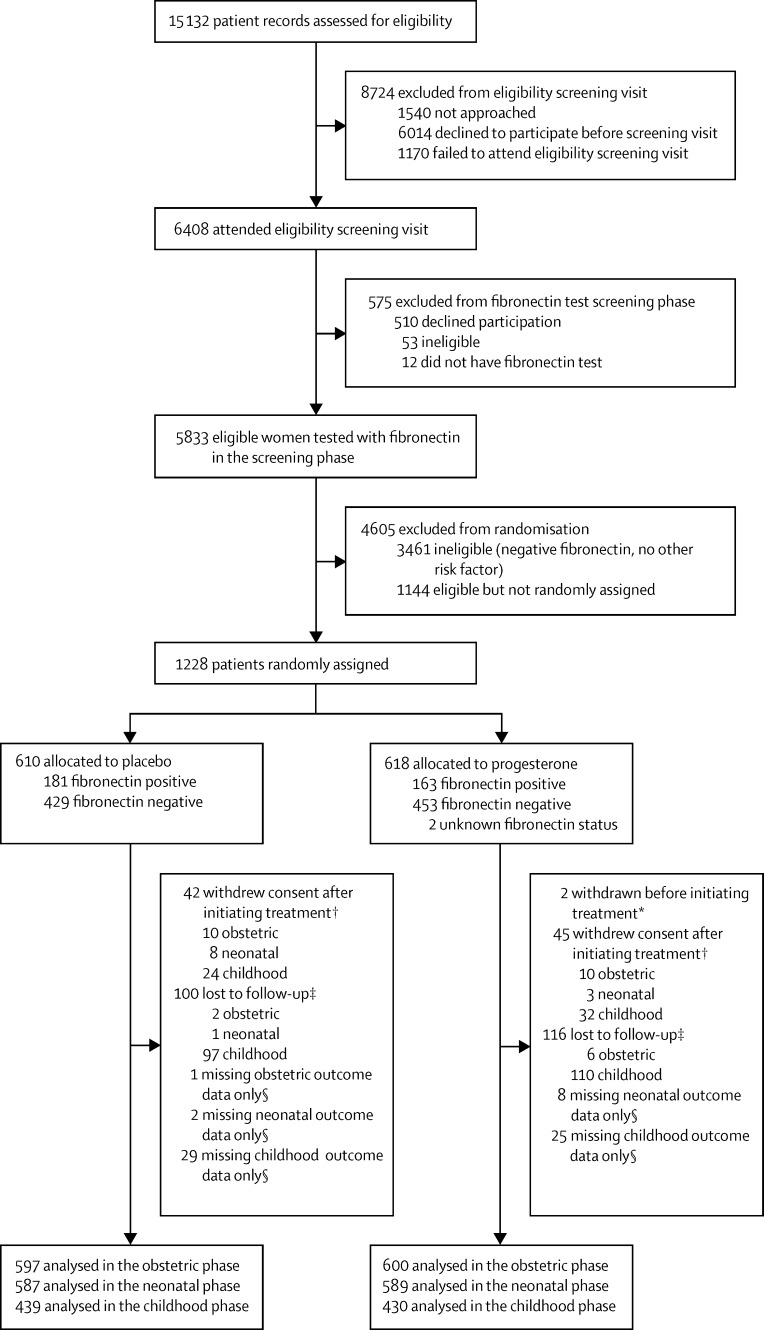
Trial profile *Randomised in error, ineligible for treatment, and excluded post-randomisation. †Consent withdrawals for each of the phases refer to consent withdrawal at any time before reaching the outcome for that phase. ‡Losses to follow-up for each of the phases refer to losses to follow-up at any time before reaching the outcome for that phase. §Numbers with missing outcome data refer to each specific outcome only (obstetric, neonatal, and childhood) and are not additive across the stages since women can have outcome data for a later outcome.

**Table 1:** Demographics and baseline characteristics of women entered into the treatment phase of the OPPTIMUM study

		**Placebo group**		**Progesterone group**	
		**N**	**n (%) or mean (SD)**	**N**	**n (%) or mean (SD)**
Age (years)		610	31·4 (5·8)	615	31·5 (5·6)
Smoking		607	125 (21%)	613	111 (18%)
Alcohol		609	34 (6%)	614	29 (5%)
Drug use		609	9 (1%)	614	8 (1%)
Years in full-time education		568	13·5 (3·0)	554	13·5 (3·1)
Ethnic group					
	White	609	446 (73%)	615	449 (73%)
	Black	609	95 (16%)	615	85 (14%)
	Asian	609	51 (8%)	615	53 (9%)
	Mixed	609	12 (2%)	615	16 (3%)
	Other	609	5 (1%)	615	12 (2%)
Height (cm)		607	163·6 (6·4)	614	163·5 (6·7)
Weight (kg)		607	71·4 (16·7)	614	71·9 (17·5)
Body-mass index (kg/m^2^)		607	26·7 (6·1)	614	26·9 (6·4)
Systolic blood pressure (mm Hg)		608	112·4 (12·2)	611	111·3 (12·5)
Diastolic blood pressure (mm Hg)		608	66·2 (8·6)	611	65·7 (8·5)
Any previous pregnancy		609	581 (95%)	615	591 (96%)
Previous pregnancy of at least 14 weeks		609	571 (94%)	615	578 (94%)
History of preterm birth (any)		608	473 (78%)	615	493 (80%)
History of spontaneous preterm birth		598	448 (75%)	605	473 (78%)
History of livebirth followed by neonatal death		609	85 (14 %)	615	80 (13%)
History of stillbirth		609	48 (8%)	615	47 (8%)
Cervical length		351	28·8 (11·1)	361	28·2 (10·6)
	Cervical length ≤25 mm	351	119 (34%)	361	137 (38%)
	Cervical length ≤15 mm	351	47 (13%)	361	51 (14%)
Fibronectin testing in screening phase					
	Gestation (weeks) at fibronectin test	610	22·9 (0·6)	615	22·9 (0·6)
	Positive fibronectin test result	610	180 (30%)	615	163 (27%)

Continuous variables are mean (SD), categorical variables are n (%).

**Table 2:** Primary outcomes and their components for women entered into the treatment phase of the OPPTIMUM study and their babies

		**Placebo group**	**Progesterone group**	**Unadjusted odds ratio (95% CI) or difference in means (95% CI)**	**p value (unadjusted)**	Adjusted odds ratio (95% CI)*or difference in means (95% CI)	p value (adjusted*)
Fetal death or delivery <34 weeks of gestation		108/597 (18%)	96/600 (16%)	0·86 (0·64 to 1·17)	0·34	0·86 (0·61 to 1·22)	0·67
Neonatal morbidity or death		60/587 (10%)	39/589 (7%)	0·62 (0·41 to 0·94)	0·02	0·62 (0·38 to 1·03)	0·072
Cognitive composite score at 2 years†‡		97·7 (17·5)	97·3 (17·9)	−0·48 (−2·77 to 1·81)§	0·68	−0·48 (−2·77 to 1·81)§	0·68
Components of the obstetric outcome							
	Fetal death	7/597 (1%)	8/600 (1%)	1·14 (0·41 to 3·17)	0·8	..	..
	Liveborn delivery before 34 weeks	101/590 (17%)	88/592 (15%)	0·85 (0·62 to 1·15)	0·29	..	..
Components of the neonatal outcome							
	Neonatal death	6/597 (1%)	1/600 (<1%)	0·17 (0·06 to 0·49)	0·0009¶	..	..
	Bronchopulmonary dysplasia‖	18/574 (3%)	17/580 (3%)	0·94 (0·49 to 1·78)	0·84	..	..
	Brain injury on ultrasound scan**	34/574 (6%)	18/584 (3%)	0·50 (0·31 to 0·84)	0·008	..	..

Binary outcomes are n/N (%) and continuous outcomes are mean (SD).

* CI for odds ratio (OR) and p value adjusted for multiple primary outcomes using Bonferroni-Holm method.

† Median weeks of age at assessment: 111·6 weeks (IQR 104·6–122·2) in the placebo group and 110·4 weeks (104·0–121·5) in the progesterone group.

‡ Sample size of 439 in the placebo group and 430 in the progesterone group and includes imputations for deaths.

§ Difference in means (95% CI).

¶ Unadjusted for previous pregnancy of at least 14 weeks because of small sample size.

‖ Bronchopulmonary dysplasia defined as need for at least 30% oxygen to maintain oxygen saturation above 92% or positive pressure (positive pressure ventilation or nasal continuous positive airway pressure) at 36 weeks postmenstrual age or discharge, whichever comes first.

** Brain injury on ultrasound scan defined as any intraventricular haemorrhage (excludes subependymal haemorrhages), parenchymal cystic or haemorrhagic lesion, or persistent ventriculomegaly (ventricular index >97th percentile); the components of the brain scan abnormalities were: intraventricular haemorrhage 13 (3%) of 383 patients and seven (2%) of 357 patients, parenchymal cystic or haemorrhagic lesion 23 (6%) of 382 and eight (2%) of 357, and persistent ventriculomegaly (>97th percentile) eight (2%) of 372 and three (1%) of 349 in the placebo group and the progesterone group, respectively.

**Table 3:** Prespecified subgroup analyses based on baseline risk factors in women entered into the treatment phase of the OPPTIMUM study

		**Treatment effect**				***p*_interaction_**
		OR or mean difference (95% CI); p value	N	OR or mean difference (95% CI); p value	N	
Fibronectin status		Negative	Negative	Positive	Positive	
	Obstetric outcome	0·88 (0·58 to 1·33); 0·542	859	0·91 (0·57 to 1·46); 0·707	338	0·91
	Neonatal outcome	0·65 (0·37 to 1·13); 0·129	847	0·64 (0·34 to 1·20); 0·162	329	0·96
	Childhood outcome	−0·63* (−3·28 to 2·03); 0·644	628	−1·09* (−5·41 to 3·23); 0·612	241	0·86
Cervical length at baseline		>25 mm	>25 mm	≤25 mm	≤25 mm	
	Obstetric outcome	0·88 (0·50 to 1·57); 0·672	445	0·69 (0·39 to 1·20); 0·191	251	0·54
	Neonatal outcome	0·74 (0·35 to 1·56); 0·432	436	0·54 (0·25 to 1·16); 0·113	246	0·56
	Childhood outcome	−2·27* (−6·10 to 1·56); 0·247	317	−2·15* (−7·23 to 2·93); 0·408	179	0·97
Cervical length at baseline		>15 mm	>15 mm	≤15 mm	≤15 mm	
	Obstetric outcome	0·77 (0·48 to 1·23); 0·274	599	0·91 (0·41 to 2·04); 0·819	97	0·73
	Neonatal outcome	0·73 (0·39 to 1·38); 0·334	588	0·49 (0·18 to 1·31); 0·156	94	0·50
	Childhood outcome	−2·49* (−5·77 to 0·78); 0·137	423	−0·69* (−8·60 to 7·22); 0·865	73	0·68
Chorioamnionitis		No	No	Yes	Yes	
	Obstetric outcome	1·38 (0·55 to 3·45); 0·497	115	2·17 (0·68 to 6·85); 0·190	57	0·55
	Neonatal outcome	0·81 (0·22 to 2·96); 0·752	115	2·21 (0·76 to 6·40); 0·148	56	0·24
	Childhood outcome	−2·30* (−10·30 to 5·70); 0·575	81	−1·08* (−11·91 to 9·76); 0·846	43	0·86
History of spontaneous preterm birth		No	No	Yes	Yes	
	Obstetric outcome	0·99 (0·51 to 1·92); 0·972	273	0·82 (0·58 to 1·16); 0·254	903	0·62
	Neonatal outcome	1·22 (0·55 to 2·71); 0·620	270	0·48 (0·29 to 0·79); 0·0042	886	0·053
	Childhood outcome	−1·11* (−5·96 to 3·73); 0·653	201	−0·14* (−2·79 to 2·52); 0·919	656	0·73
History of any preterm birth		No	No	Yes	Yes	
	Obstetric outcome	1·06 (0·53 to 2·12); 0·868	250	0·81 (0·58 to 1·14); 0·225	946	0·50
	Neonatal outcome	1·09 (0·48 to 2·45); 0·836	248	0·52 (0·32 to 0·84); 0·0079	927	0·12
	Childhood outcome	−0·91* (−5·92 to 4·11); 0·724	187	−0·37* (−2·96 to 2·23); 0·782	681	0·85

Logistic or linear mixed effects regression models predicting outcome from treatment, subgroup and the interaction of treatment with the subgroup variable, adjusting for previous pregnancy of at least 14 weeks and a random effect for study centre.

* Mean difference.

**Table 4:** Secondary outcomes

			**Placebo group**		**Progesterone group**		**OR, HR, or mean difference (95% CI)**	**p value**
			N	n (%) or mean (SD)	N	n (%) or mean (SD)		
**Obstetric and neonatal**								
Gestational age at delivery (weeks)			597	36·8 (4·2)	600	36·9 (4·1)	1·03 (0·92 to 1·15)	0·62
Deaths up to 2 years of age			509	16 (3%)	500	20 (4%)	1·28* (0·66 to 2·51)	0·47
Death after trial entry up to end of study			598	16 (3%)†	600	20 (3%)†	1·26* (0·65 to 2·42)	0·5
Daily category of care after delivery room								
	Number of days of normal care		570	1·7 (2·3)	581	1·7 (1·6)		
	Number of days of special care		570	4·2 (10·6)	581	2·9 (8·3)		
	Number of days of high dependency care		569	2·2 (8·4)	580	2·1 (10·4)		
	Number of days intensive care		569	1·8 (7·3)	580	1·9 (8·1)		
Surfactant administration			573	45 (8%)	583	47 (8%)	1·03 (0·68 to 1·55)	0·9
Suspected or confirmed necrotising entercolitis			574	13 (2%)	581	18 (3%)	1·37 (0·76 to 2·45)	0·29
Infections								
	Neonatal infection		573	36 (6%)	537	44 (8%)	1·22 (0·79 to 1·88)	0·36
	One or more discrete episodes with positive blood culture among those with infection		33	19 (58%)	40	17 (42%)	0·51 (0·19 to 1·34)	0·18
	One or more discrete episodes with positive CNS culture among those with infection		34	0	40	3 (7%)	‡	0·25§
Maternal or child serious adverse event during pregnancy and birth			610	70 (11%)	616	59 (10%)	0·83 (0·58 to 1·16)	0·27
**Childhood (2 years of age)**								
Health								
	Composite outcome of death or moderate-to-severe neurodevelopmental impairment at 2 years		419	51 (12%)	399	67 (17%)	1·45 (0·98 to 2·15)	0·064
	Moderate-to-severe neurodevelopmental impairment		403	35 (9%)	379	47 (12%)	1·48 (0·98 to 2·33)	0·087
	Individual components of disability							
		Motor	456	4 (1%)	461	4 (1%)	0·99¶ (0·25 to 3·98)	0·99
		Cognitive function	452	18 (4%)	461	19 (4%)	1·03 (0·58 to 1·84)	0·92
		Hearing	465	2 (<1%)	466	1 (<1%)	0·56¶ (0·33 to 0·94)	0·028
		Speech and language	446	14 (3%)	445	18 (4%)	1·32 (0·72 to 2·43)	0·36
		Vision	466	4 (1%)	447	0	‡	0·13§
		Respiratory	434	3 (1%)	413	7 (2%)	3·03¶ (1·56 to 5·88)	0·0011
		Gastrointestinal	432	4 (1%)	412	9 (2%)	2·67¶ (1·37 to 5·20)	0·004
		Renal	434	1 (<1%)	414	3 (1%)	3·65 (1·96 to 6·82)	<0·0001
	Admitted to hospital during follow-up		434	51 (12%)	416	48 (12%)	0·98 (0·65 to 1·47)	0·92
Behavioural scale scores at 2 years assessed in strengths and difficulties questionnaire‖								
	Total difficulties		302	9·8 (4·9)	295	10·2 (4·9)	1·23 (0·85 to 1·78)**‡‡	0·28
	Emotional problems		341	1·1 (1·2)	328	1·1 (1·2)	1·01 (0·61 to 1·67)**‡‡	0·96
	Conduct problems		342	2·7 (1·8)	326	2·6 (1·8)	0·92 (0·65 to 1·31)**‡‡	0·66
	Hyperactivity scale		334	4·2 (2·4)	315	4·5 (2·3)	1·10 (0·79 to 1·55)**‡‡	0·57
	Peer problems scale		345	2·0 (1·7)	318	2·1 (1·6)	1·22 (0·88 to 1·69)**‡‡	0·22
	Prosocial scale		339	6·3 (2·2)	320	5·9 (2·3)	1·20 (0·88 to 1·63)**‡‡	0·25
	Impact scale		424	0·2 (1·0)	404	0·2 (1·2)	1·31 (0·73 to 2·35)**‡‡	0·37
EQ-5D								
	Change in EQ-5D from baseline to birth		199	−0·023 (0·220)	191	−0·021 (0·207)	0·001§§ (−0·034 to 0·036)	0·97
	Change in EQ-5D from baseline to 12 months		274	−0·015 (0·221)	279	−0·009 (0·213)	0·003§§ (−0·026 to 0·032)	0·83
Women's views								
	Women's perception of treatment 1 month post-delivery (proportion extremely or fairly satisfied)		327	314 (96·0)	307	294 (95·6)	0·93 (0·42 to 2·04)	0·85

* Hazard ratio (HR).

† Median time to death: 759 days (range 1–1331) in the placebo group and 751 days (1–1335) in the progesterone group.

‡ Regression failed with and without adjusting for previous pregnancy of more than 14 weeks.

§ Exact Fisher test.

¶ Not adjusted for previous pregnancy of at least 14 weeks because regression failed.

‖ Mean age at assessment: 107·7 weeks (SD 17·7) in the placebo group and 106·9 weeks (17·1) in the progesterone group.

** Score analysed as binary variable (raised compared with normal score).

‡‡ Odds ratio (OR) of abnormal score.

§§ Mean difference.

**Table 5:** Safety outcomes

			**Placebo group**		**Progesterone group**	
			N	n (%) or mean (SD)*	N	n (%) or mean (SD)*
**Pregnancy complications**						
Maternal						
	Obstetric cholestasis		589	6 (1%)	593	4 (1%)
	Hypertension		590	24 (4%)	593	23 (4%)
	Pre-eclampsia		590	11 (2%)	593	10 (2%)
	Eclampsia		590	1 (<1%)	593	0
	Preterm premature membrane rupture		590	72 (12%)	593	65 (11%)
	Antepartum haemorrhage		590	36 (6%)	593	37 (6%)
	Gestational diabetes		590	37 (6%)	593	27 (5%)
	Confirmed deep vein thrombosis		590	2 (<1%)	593	0
	Cervical cerclage		360	39 (11%)	368	41 (11%)
	Other maternal complication		590	164 (28%)	593	166 (28%)
Fetal						
	Any		590	18 (3%)	593	19 (3%)
	Abdominal circumference <5th percentile		18	4 (22%)	19	6 (32%)
	Liquor volume reduced		18	6 (33%)	19	6 (32%)
	Doppler >95th percentile (umbilical artery)		18	1 (6%)	19	1 (5%)
	Absent end diastolic flow (umbilical artery)		18	0	19	1 (5%)
	Reversed end diastolic flow (umbilical artery)		18	1 (6%)	19	1 (5%)
	Abnormal antenatal CTG		18	7 (39%)	19	3 (16%)
Hospital admissions						
	Antenatal hospital admissions per woman					
		Mean (SD)	581	0·7 (1·3)	579	0·6 (1·1)
		Median (range)	581	0 (0–10)	579	0 (0–8)
	Hospital admissions for threatened preterm labour		581	132 (23%)	579	119 (21%)
		With tocolysis	581	18 (3%)	579	15 (3%)
		With steroid	581	71 (12%)	579	80 (14%)
		With antibiotic	581	52 (9%)	579	38 (7%)
		With cervical cerclage	581	10 (2%)	579	8 (1%)
		With magnesium sulfate	581	0	579	0
	Women with antenatal hospital admission for other reasons		581	135 (23%)	579	107 (18%)
Labour						
	Duration of first stage (h)		463	4·1 (5·1)	470	4·3 (5·3)
	Duration of second stage (min)		462	47·0 (132·8)	471	41·2 (91·6)
	Duration of third stage (min)		465	17·0 (46·2)	477	16·1 (51·6)
Artificial rupture of membranes performed			468	131 (28%)	448	122 (27%)
Analgesia in labour (any)			576	455 (79%)	574	478 (83%)
	General anaesthetic		576	16 (3%)	574	12 (2%)
	Epidural		576	191 (33%)	574	197 (34%)
	Opiates		576	88 (15%)	574	88 (15%)
	Nitrous oxide		576	269 (47%)	574	303 (53%)
	Other		576	34 (6%)	574	31 (5%)
Delivery method						
	Spontaneous vaginal delivery		578	380 (66%)	576	375 (65%)
	LSCS in labour		578	58 (10%)	576	57 (10%)
	LSCS pre-labour		578	92 (16%)	576	84 (15%)
	Forceps		578	21 (4%)	576	27 (5%)
	Ventouse		578	18 (3%)	576	20 (3%)
	Vaginal breech (spontaneous or assisted)		578	9 (2%)	576	13 (2%)
Blood loss (mL)			572	387 (356)	572	424 (394)
Blood transfusion			578	10 (2%)	574	18 (3%)
Antibiotics during labour and delivery			578	96 (17%)	573	92 (16%)
Surgical procedure required			578	15 (3%)	575	18 (3%)
Mean duration of hospital stay (days)			577	3·2 (2·2)	567	3·3 (4·1)
Median duration of hospital stay, days (range)			577	3·0 (1·0–19·0)	567	3·0 (1·0–86·0)
Any post-partum complication			580	83 (14%)	577	90 (16%)
Placental examination						
	No evidence of infection		84	57 (68%)	83	56 (67%)
	Chorioamnionitis		84	10 (12%)	83	9 (11%)
	Chorioamnionitis and funisitis		84	17 (20%)	83	18 (22%)
**Birth outcomes**						
Male sex†			578	289 (50%)	578	293 (51%)
Birthweight (g)			577	2822 (884)	577	2875 (847)
Median Apgar score at 1 min (IQR)			553	9·0 (8·0–9·0)	557	9·0 (8·0–9·0)
Median Apgar score at 5 min (IQR)			555	9·0 (9·0–10·0)	560	9·0 (9·0–10·0)
Median length of hospital stay, days (IQR)			556	2·0 (1·0–6·0)	562	2·0 (1·0–4·0)
**Outcomes at 2 years**						
Weight (kg)			355	13·2 (2·6)	332	13·4 (2·7)
Height (cm)			369	87·2 (10·7)	347	87·4 (7·9)
Head circumference (cm)			354	48·9 (4·6)	332	49·6 (6·7)

* Data are n (%) or mean (SD), unless otherwise stated. Outcomes in the safety population (ie, women who took at least one tablet of placebo or progesterone). CTG=cardiotocograph. LSCS=lower segment caesarean section.

† One baby of indeterminate sex in the progesterone group.
