# Department of Error

**DOI:** 10.1016/S0140-6736(18)32625-4

**Published:** 2019-01-19

*Norman JE, Marlow N, Messow C-M, et al, for the OPPTIMUM study group. Vaginal progesterone prophylaxis for preterm birth (the OPPTIMUM study): a multicentre, randomised, double-blind trial.* Lancet *2016; **387:** 2106–16*—In this Article, ten neonatal deaths (eight in the progesterone and two in the control group) were incorrectly categorised as postneonatal deaths, with no effect on the primary outcome and study conclusions. These corrections have been made online as of Jan 17, 2019, and the supplementary appendix of this Article has also been corrected as of Jan 17, 2019.

