# Department of Error

**DOI:** 10.1016/S0140-6736(19)30860-8

**Published:** 2019-04-20

*Norman JE, Marlow N, Messow C-M, et al, for the OPPTIMUM study group. Vaginal progesterone prophylaxis for preterm birth (the OPPTIMUM study): a multicentre, randomised, double-blind trial.* Lancet *2016; **387:** 2106–16—*In this Article, in table 2, the upper 95% CI of the unadjusted odds ratio for the primary outcome of neonatal morbidity or death should be 1·07; in table 3, the p value of the treatment effect in the neonatal outcome in women with history of spontaneous preterm birth should be 0·033. These corrections have been made to the online version as of April 18, 2019.

