# Supplementary material for: Vaginal progesterone prophylaxis for preterm birth (the OPPTIMUM study): a multicentre, randomised, double-blind trial
Source: Lancet. 2016 May 21;387(10033):2106–16. doi: 10.1016/S0140-6736(16)00350-0 (PMC5406617; doi:10.1016/S0140-6736(16)00350-0)
Supplement: Supplementary appendix [file mmc1.pdf]

THE LANCET

Supplementary appendix

This appendix formed part of the original submission and has been peer reviewed. We post it as supplied by the authors.

Supplement to: Norman JE, Marlow N, Messow C-M, et al, for the OPPTIMUM study group. Vaginal progesterone prophylaxis for preterm birth (the OPPTIMUM study): a multicentre, randomised, double-blind trial. *Lancet* 2016; published online Feb 23. http://dx.doi.org/10.1016/S0140-6736(16)00350-0.

Supplementary Figure 1: Eligibility and allocation to fFN (fibronectin) positive and ffN negative groups in the treatment phase.

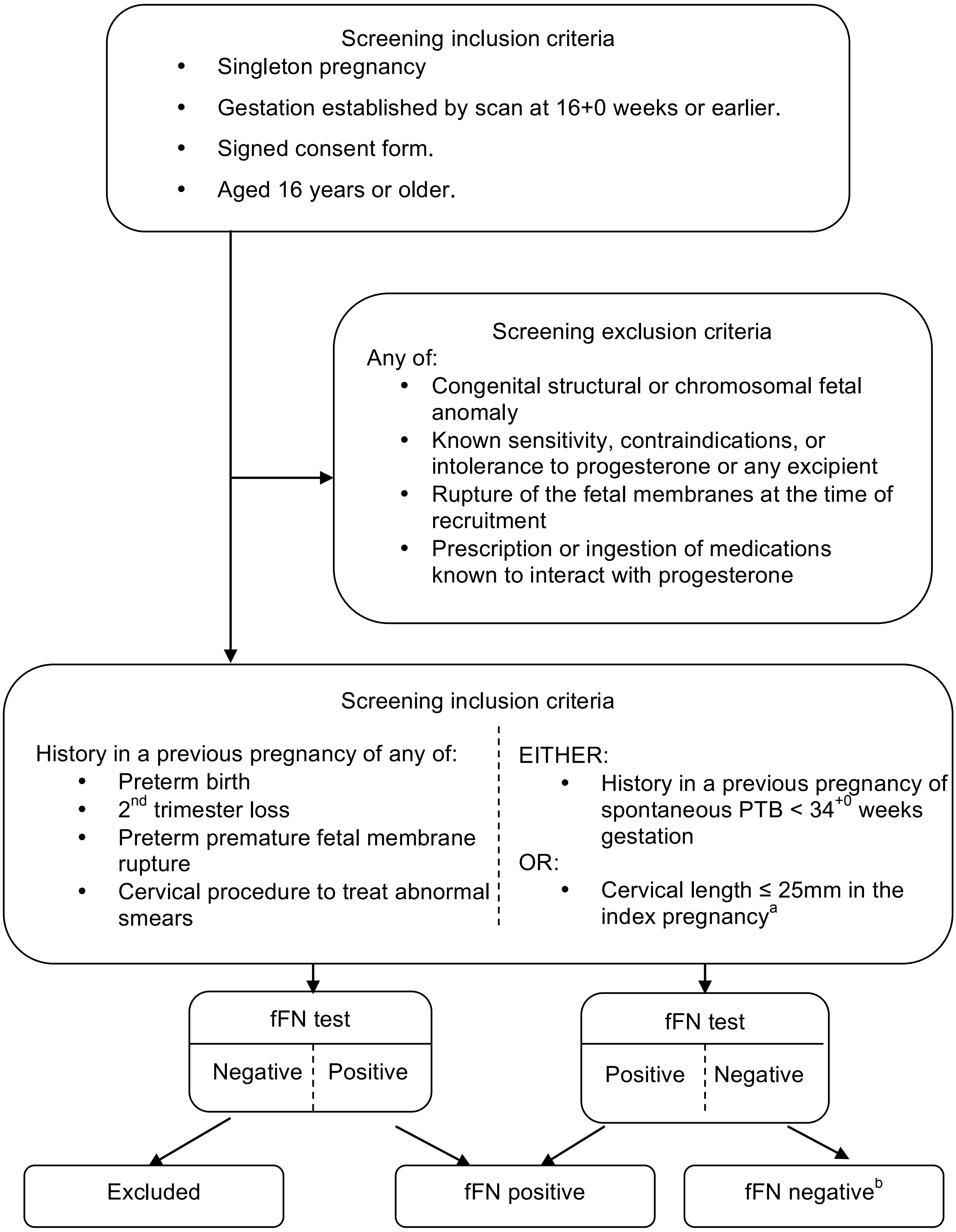

[a]Measured at between 18^{+0} and 24^{+0} weeks gestation
[b]Eligible from September 2010 onwards

Supplementary Figure 2 Survival curve for gestation at delivery

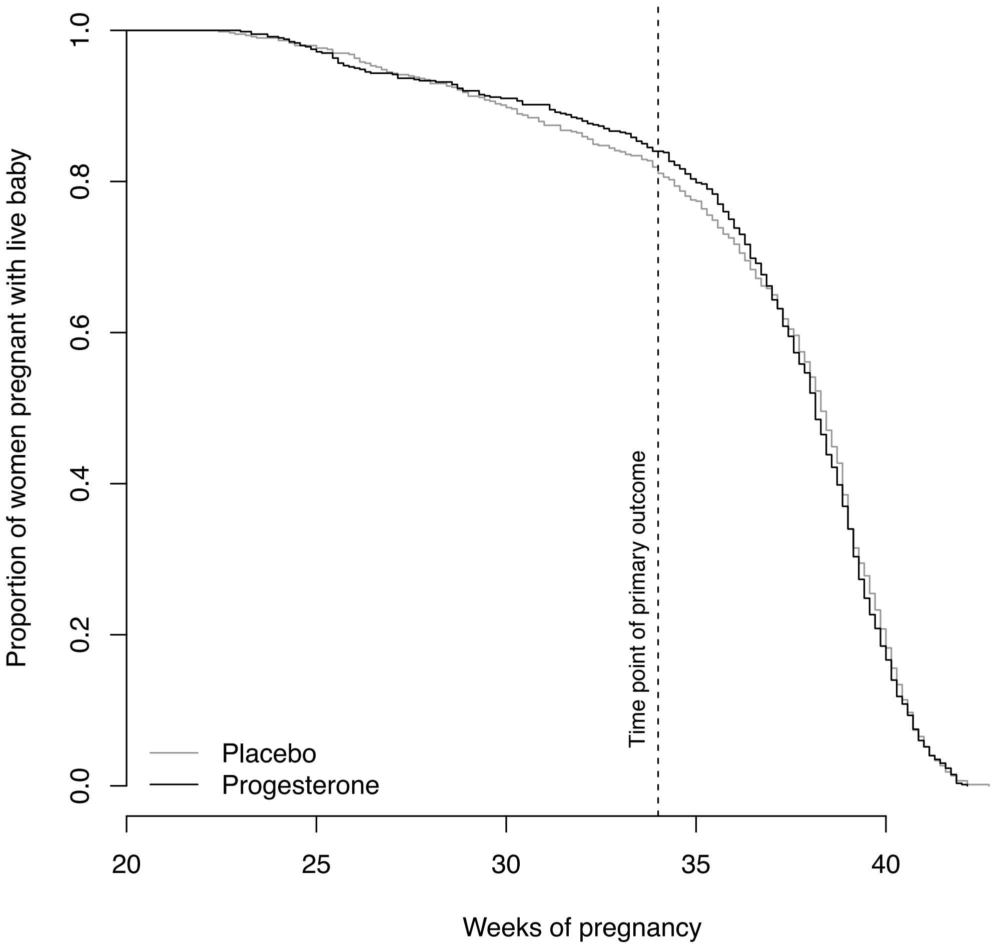

Supplementary Table 1.

Characteristics of those participants for whom a childhood outcome was and was not available.

**Characteristic**	**No childhood outcome available (N=356)**	**Childhood outcome available (N=869)**
**General**		
Age - years	29.6 ± 5.7	32.2 ±5.5
Smoking – N (%)	110 (31.0%)	126 (14.6%)
Alcohol- N (%)	21 (5.9%)	42 (4.8%)
Drug use - N (%)	8 (2.2%)	9 (1.0%)
Years in full-time education[a]	12.8 ± 2.8	13.8 ± 3.1
Ethnic group		
- White	254 (71.3%)	641 (73.8%)
- Black	62 (17.4%)	118 (13.6%)
- Asian	27 (7.6%)	77 (8.9%)
- Mixed	8 (2.2%)	20 (2.3%)
- Other	5 (1.4%)	12 (1.4%)
Height - cm	163.6 ± 6.6	163.5 ± 6.6
Weight - kg	70.3 ± 15.7	72.2 ± 17.6
BMI (kg/m^2)	26.3 ± 5.5	27.0 ± 6.5
Systolic Blood Pressure - mmHg [b]	111.1 ± 12.0	112.2 ± 12.5
Diastolic Blood Pressure - mmHg [b]	65.4 ± 8.8	66.2 ± 8.5
**Obstetric history**		
Any previous pregnancy - N (%)	345 (97.2%)	827 (95.2%)

previous pregnancy of at least 14 weeks - N (%)	337 (94.9%)	812 (93.3%)
History of preterm birth (any) - N (%)	285 (80.3%)	681 (78.5%)
spontaneous preterm birth - N (%)[d]	265 (76.6%)	656 (76.5%)
History of live birth followed by neonatal death - N (%)	44 (12.4%)	121 (13.9%)
History of stillbirth -N (%)	29 (8.2%)	66 (7.6%)
**Cervical length – mm[c]**	28.4 ± 10.6	28.6 ± 11.0
Cervical length ≤25mm[c] - N (%)	77 (35.6%)	179 (36.1%)
Cervical length ≤15mm [c] - N (%)	25 (11.6%)	73 (14.7%)
**Fibronectin testing in screening phase**		
Gestation (weeks) at fFN test	22.9 (0.6)	22.9 (0.6)
Positive fFN test result - N (%)	103 (28.9%)	241 (27.7%)

Continuous variables are summarised as mean ± standard deviation (plus-minus values), categorical variables as number (percentage) per category. All variables are missing for a maximum of 5 women in each group, except where specified otherwise.

[a] Missing for 24 / 29 women in childhood outcome unavailable / available respectively.

[b] Missing for 1 / 6 women in childhood outcome unavailable / available respectively

[c] Missing for 141 / 373 women in childhood outcome unavailable / available respectively

[d] Missing for 11 / 12 women in childhood outcome unavailable / available respectively

Supplementary Table 2. Per protocol analyses of primary outcome

Outcome	OR	95% CI	P value	Adjusted P value*
Obstetric – death or preterm birth < 34 weeks n (%)	0.86	0.55, 1.35	0.51	1.00
Neonatal - composite of death, brain injury or severe CLD) n (%)	0.55	0.30, 0.99	0.046	0.14
	Treatment Effect	95% CI	P value	
Childhood – mean between-group difference in Bayley III at 2 years - cognitive composite score including imputations for deaths,	0.49	-2.22, 3.20	0.73	0.73

* Adjusted p-values are given to facilitate comparison with results in the ITT population.

Supplementary Table 3. Analysis performed with multiple imputations of primary outcome

Outcome	Parameter estimate or Hazard Ratio	95% confidence interval	p-value
Obstetric[a] outcome	0.866	0.640, 1.170	0.35
Neonatal[a] outcome	0.637	0.418, 0.971	0.036
Childhood outcome[b]	-0.019	-0.372, 0.334	0.91
Childhood outcome[c]	-0.051	-0.371, 0.269	0.74

Ten imputations were run.

[a] Variables used for predicting outcome: Previous pregnancy of at least 14 weeks, high/low risk, maternal age, sex.

[b] Variables used for predicting outcome: Gestational age, birth weight, Chronic Lung Disease, brain injury, previous pregnancy of at least 14 weeks, high/low risk, maternal age, sex.

[c] Variables used for predicting outcome: Birth weight, Chronic Lung Disease, brain injury, previous pregnancy of at least 14 weeks, high/low risk, maternal age, sex.

Note, for comparison with primary analysis: Adjusted p-values are: obstetric outcome p= 0.11, neonatal outcome p= 0.7, childhood outcome remains unchanged.

Supplementary Table 4

Characteristics of those participants who were and who were not included in the per protocol analysis.

**Characteristic**	**Not included in per protocol analysis (N=539)**	**Included in per protocol analysis (N=687)**
**General**		
Age - years	30.5 ± 5.7	32.2 ± 5.5
Smoking – N (%)	137 (25.7%)	99 (14.4%)
Alcohol- N (%)	32 (6.0%)	31 (4.5%)
Drug use - N (%)	7 (1.3%)	10 (1.5%)
Years in full-time education[a]	13.3 ± 3.1	13.6 ± 3.0
Ethnic group		
- White	374 (69.6%)	521 (75.8%)
- Black	97 (18.1%)	83 (12.1%)
- Asian	46 (8.6%)	58 (8.4%)
- Mixed	13 (2.4%)	15 (2.2%)
- Other	7 (1.3%)	10 (1.5%)
Height - cm	163.8 ± 6.8	163.3 ± 6.4
Weight - kg	71.7 ± 16.8	71.6 ± 17.3
BMI (kg/m^2)	26.7 ± 6.1	26.8 ± 6.4
Systolic Blood Pressure - mmHg	111.6 ± 12.4	112.0 ± 12.4
Diastolic Blood Pressure - mmHg	65.9 ± 9.0	66.0 ± 8.2
**Obstetric history**		

Any previous pregnancy - N (%)	525 (97.8%)	647 (94.2%)
previous pregnancy of at least 14 weeks - N (%)	515 (95.9%)	634 (92.3%)
History of preterm birth (any) - no (%)	429 (80.0%)	537 (78.2%)
spontaneous preterm birth - N (%)[c]	404 (77.0%)	517 (76.3%)
History of live birth followed by neonatal death - N (%)	73 (13.6%)	92 (13.4%)
History of stillbirth -N (%)	55 (10.2%)	40 (5.8%)
**Cervical length – mm[b]**	28.9 ± 10.7	28.2 ± 11.0
Cervical length ≤25mm[b] - N (%)	114 (34.7%)	142 (37.1%)
Cervical length ≤15mm [b] - N (%)	43 (13.1%)	55 (14.4%)
**Fibronectin testing in screening phase**		
Gestation (weeks) at fFN test	22.9 (0.7)	22.9 (0.6)
Positive fFN test result – N (%)	152 (28.2%)	192 (27.9%)

Continuous variables are summarised as mean ± standard deviation (plus-minus values), categorical variables as number (percentage) per category. All variables are missing for a maximum of 5 women in each group, except where specified otherwise.

[a] Missing for 59 / 44 women in excluded and included in per protocol analysis respectively.

[b] Missing for 210/304 women in excluded and included in per protocol analysis respectively

[c] Missing for 14/9 women in excluded and included in per protocol analysis respectively

Supplementary Table 5 Rates of preterm birth in subgroups

**Supplementary table** Primary obstetric outcome in subgroups.		
Risk group	Placebo	Progesterone
	Number with event / number with data available (percentage).	
Fibronectin status		
Fibronectin negative	54 / 418 (12.9)	51 / 442 (11.5)
Fibronectin positive	54 / 179 (30.2)	45 / 159 (28.3)
Cervical length at baseline		
> 25mm	29 / 228 (12.7)	25 / 217 (11.5)
≤ 25mm	38 / 118 (32.2)	33 / 133 (24.8)
Cervical length at baseline		
> 15mm	46 / 299 (15.4)	37 / 300 (12.3)
≤ 15mm	21 / 47 (44.7)	21 / 50 (42.0)
History of spontaneous preterm birth		
No	23 / 149 (15.4)	19 / 124 (15.3)
Yes	82 / 437 (18.8)	74 / 466 (15.9)
History of any preterm birth		
No	20 / 135 (14.8)	18 / 115 (15.7)
Yes	88 / 461 (19.1)	78 / 485 (16.1)

Supplementary text 1: TSC and DMC membership

The OPPTIMUM investigators are very grateful to the following for their participation in the Trial Steering and Data Monitoring Committees.

Trial Steering Committee:

John Morrison (Chair) National University of Ireland, Galway, Ireland; Denis Azzopardi, Imperial College, London, UK; Jane Brewin, Tommy's, the Baby Charity, London, UK; John M Davison, The Medical School, University of Newcastle, Newcastle, UK; Sara Kenyon, University of Birmingham, Birmingham, UK; Gillian Pillans, Lay representative, England, UK. Maggie Redshaw, NPEU, Oxford, UK.

Data Monitoring Committee: Henry Halliday (Chair) Royal Maternity Hospital, Belfast, Northern Ireland, UK; Simon Gates University of Warwick, Coventry, UK; Gerard H.A. Visser, University Medical Centre, Utrecht, Netherlands

Supplementary Text 2

Other acknowledgements.

We are grateful to the following for their help in the conduct of the OPPTIMUM study: OPPTIMUM trial staff: Lorraine Adamson, Trial Administrator, University of Edinburgh, Edinburgh, UK; Amy Barker, St. Thomas Hospital, London, UK; Tariq Derdeb, Trial Administrator, University of Edinburgh, Edinburgh, UK; Kasia Adamczuk, Assistant Clinical Trial Manager, University of Edinburgh, Edinburgh, UK; Bernadette Gallagher, Assistant Clinical Trial Manager, University of Edinburgh, Edinburgh, UK; Christine Hill-Evans, Trial Co-ordinator, University Hospital of Coventry, Coventry, UK; Girija Kadlaskar, Paediatric Assessor, University College Hospital, London, UK; Marzena Orzol, Follow-up Co-ordinator /Paediatric Assessor, University College Hospital, London, UK; Ateeka Poole, Follow-up Co-ordinator /Paediatric Assessor, University College Hospital, London, UK; Faye Sutton, Trial Co-ordinator, University of Exeter Medical School, Exeter, UK; Amy Witherspoon, Trial Administrator, University of Edinburgh, Edinburgh, UK.

Staff at the Robertston Centre for Biostatistics: Jane Aziz, Senior Application Programmer, University of Glasgow, Glasgow, UK; Lorna Gillespie, Senior Clinical Database Manager, University of Glasgow, Glasgow, UK; Sharon Kean, Director, Information Systems, University of Glasgow, Glasgow, UK.

Bayley assessors: Aiwyne Foo, Chesterfield Royal Hospital, Chesterfield, UK; Alison Kimber, Royal Victoria Infirmary, Newcastle University Teaching Hospitals, Newcastle, UK; Cassie Lawn, Brighton & Sussex University Hospitals, Brighton, UK; Christine Cornforth, Liverpool Women's NHS Foundation Trust, Liverpool, UK; Hilary Cruickshank, Royal Infirmary of Edinburgh, Edinburgh, UK; Joyce Cummings, NHS Fife, Kirkcaldy, UK; Tara Fairley, Aberdeen Maternity Hospital, Aberdeen, UK; Julia Goodwin, Basingstoke & North Hampshire Hospital, Basingstoke, UK; Julia Halpin, Royal Devon & Exeter Hospitals NHS Trust, Exeter, UK; Megan Thomas, Blackpool Teaching Hospitals, Blackpool, UK; Patricia Dulson, Newcastle University Teaching Hospitals, Newcastle, UK; Sridhar Kalyanasundaram, Wishaw General Hospital, Wishaw, UK; Rachel Liddell, NHS Greater Glasgow & Clyde, Glasgow, UK;

Samantha Parry, Liverpool Women's NHS Foundation Trust, Liverpool, UK; Gargeswari Sunanda, Heart of England Hospital, Birmingham, UK

Other investigators and recruiting midwives:

Lilian Alabi-Isama, Imperial College London, London, UK; Agneta Cedefors-Blom, Research Coordinator, Sahlgrenska University Hospital, Gothenburg, Sweden; Joanna Cook, Co-Investigator, Queen Charlotte’s Hospital, Imperial College Healthcare NHS Trust, London, UK; Fiona Denison, Co-Investigator, Royal Infirmary of Edinburgh, Edinburgh, UK; Jon Dorling, Paediatrician, QMC & City Hospital , Nottingham University Hospitals NHS Trust, Nottingham, UK; Medhat Ezzat, Neonatologist, Aberdeen Maternity Hospital, Aberdeen, UK; Luca Fusi, Ealing Hospital, London North West Healthcare NHS Trust, London, UK; Andrew Gallagher, Paediatrician, Worcestershire Royal Hospitals NHS Trust, Worcester, UK; Natalie Greenwold, Co-Investigator, University College Hospital, London, UK; Bibbi Hagberg, Clinical Psychologist, Gillberg Neuropsychiatry Centre, Sahlgrenska Academy at University of Gothenburg, Sweden; Maria Hallingström, Sahlgrenska University Hospital, Gothenburg, Sweden; Mohammed Houda, Co-Investigator, Queen’s Medical Centre, Nottingham, UK; Carlotta Iacovella, Co-Investigator, St George's Hospital, London, UK; Asma Khalil, Co-Investigator, St George's Hospital, London, UK; Lindsay Kindiger, Co-Investigator, St Mary's Hospital, London (with Imperial), London, UK; Gopi Menon, Neonatologist, Royal Infirmary of Edinburgh, Edinburgh, UK; Samundra Mukherjee, Paediatrician, Basildon & Thurrock University Hospital, Basildon, UK; Paul Munyard, Royal Cornwall Hospital, Truro, UK; Ediri O'Brien, Research Fellow, Liverpool Women's Hospital/Liverpool Women's Foundation Trust, Liverpool, UK; Gabriel Okugbeni, Co-Investigator, South Tyneside NHS Foundation Trust, South Shields, UK; Heike Rabe, Neonatologist, Brighton & Sussex University Hospitals, Brighton, UK; Amy Robb, Co-Investigator, Royal Infirmary of Edinburgh, Edinburgh, UK; Ashalatha Shetty, Co-Investigator, Aberdeen Maternity Hospital, Aberdeen, UK; Marie Smith, Co-Investigator, Royal Victoria Infirmary, Newcastle University Teaching Hospitals, Newcastle, UK; Richard Smith, Co-Investigator, Norfolk and Norwich University Hospitals, Norwich, UK; Naharmal B Soni, Co-Investigator, Burnley General Hospital, Burnley, UK; Fatimah Soydemir, Co-Investigator, Royal Preston Hospital, Preston, UK; Fatimah Soydemir,

Royal Preston Hospital, Preston, UK; Ayman Swidan, Co-Investigator, Queen's Hospital, Burton-on-Trent, UK; Lynne Sykes, Queen Charlotte's Hospital London (with Imperial), London, UK; Arumugavelu Thirumurugan, Paediatrician, Leighton Hospital, Mid Cheshire Hospitals NHS Foundation Trust, Crewe, Cheshire, UK; Leena Tripathi, Co-Investigator, Blackpool Teaching Hospital, Blackpool, UK; Melissa Whitworth, Co-Investigator, St Mary's Hospital (Manchester), Manchester, UK; Mahesh Yadav, Neonatologist, Royal Bolton Hospital, Bolton, UK.

Christine Adamson, West Middlesex Hospital, London, UK; Helen Ayre, Lincoln County Hospital, Lincoln, UK; Sarah Bailey, Princess Anne Hospital, Southampton, UK; Jackie Barlow, Royal Bolton Hospital, Bolton, UK; Annette Briley, Guy's & St Thomas's Hospital, London, UK; Jane Brooks, Blackpool Victoria Hospital, Blackpool, UK; Janet Brown, Leighton Hospital, Crewe, UK; Karen Burns, Royal Lancaster Infirmary, University Hospitals of Morecambe Bay, Lancaster, UK; Julie Butler, Royal Preston Hospital, Preston, UK; Gillian Butterfield, Bradford Teaching Hospitals NHS Trust, Bradford, UK; Jenny Carter, St Thomas's Hospital, London, UK; Rachel Crone, Warrington and Halton Hospitals NHS Foundation Trust, Warrington, UK; Yvette Davis, QMC, Nottingham University Hospitals NHS Trust, Nottingham, UK; Jennifer Devlin, Royal Infirmary of Edinburgh, Edinburgh, UK; Caroline Dixon, Leighton Hospital, Crewe, UK; Michelle Dower, Liverpool Women's NHS Foundation Trust, Liverpool, UK; Frances Dunleavy (neé Fraser), Norfolk and Norwich University Hospitals, Norwich, UK; Julie Eaton, Royal Stoke University Hospital, Stoke-on-Trent, UK; Tracey Edey (neé Lawrence), University Hospital Wales, Cardiff, UK; Diane Farrar, Bradford Royal Infirmary, Bradford, UK; Andrea Fenn, Royal Victoria Infirmary, Newcastle University Teaching Hospitals, Newcastle, UK; Judy Filmer, St Thomas's Hospital, London, UK; Jane Forbes, Princess Anne Hospital, Southampton, UK; Frida Forya, University College Hospital, London, UK; Hilary Goodman, Basingstoke Hospital, Basingstoke, UK; Kerry Goodsell, Basildon and Thurrock University Hospital, Basildon, UK; Nicky Grace, City Hospital, Nottingham University Hospitals NHS Trust, Nottingham, UK; Elaine Gregor, Wishaw General Hospital, Wishaw, UK; Julie Grindey, Arrowe Park Hospital, Wirral University Teaching Hospitals NHS Foundation Trust, Wirral, UK; Yvette Gunn, QMC, Nottingham University

Hospitals NHS Trust, Nottingham, UK; Kelda Fulliano (neé Hargreaves), Norfolk and Norwich University Hospitals, Norwich, UK; Katherine Holland, Royal Cornwall Hospital, Truro, UK; Heidi Hollands, Derriford Hospital, Plymouth, UK; Helen Howlett, North Tyneside General Hospital, North Shields, UK; Suzanne Jerreat, Royal Stoke University Hospital, Stoke-on-Trent, UK; Dawn Kelly, Joanna Hutchinson, Newham University Hospital, London, UK; Worcestershire Royal Hospitals NHS Trust, Worcester, UK; Mary Kelly-Baxter, Chesterfield Royal Hospital, Chesterfield, UK; Kathryn Kershaw, Calderdale Royal Hospital, Calderdale & Huddersfield NHS Foundation Trust, Huddersfield, UK; Kari Kordtomeikel, Norfolk and Norwich University Hospitals, Norwich, UK; Tracey Lawrence, University Hospital Wales, Cardiff, UK; Ruth Leary (neé Cate), St Thomas's Hospital, London, UK; Suzanne Lee, Brighton & Sussex University Hospitals, Brighton, UK; Siobhan Limerick, Wansbeck & North Tyneside Hospital, North Shields, UK; Eleanor Lindahl, St George's Hospital, London, UK; Sandra Linton, St George's Hospital, University of London, London, UK; Julie Long, St Mary's Hospital, Newport - Isle of Wight, UK; Heather Longworth, Liverpool Women's NHS Foundation Trust, Liverpool, UK, Louise McCabe, Wishaw General Hospital, Wishaw, UK; Anna Molnar, City Hospital, Nottingham University Hospitals NHS Trust, /QMC, Nottingham University Hospitals NHS Trust, Nottingham, UK; Sally-Ann Molsher, Pilgrim Hospital, Boston, UK; Lynsey Moorhead, St Mary's Hospital, Manchester, UK; Yvonne Muwalo, St George's Hospital, London, UK; Chloe O'Hara, Birmingham Women's Hospital, Birmingham, UK; Judith Ormonde, South Tyneside NHS Foundation Trust, South Shields, UK; Rebecca Palethorpe, Bradford Teaching Hospitals NHS Trust, Bradford, UK; Kirsteen Paterson, Southern General Hospital, Glasgow, UK; Stacey Pepper, Clinical Trials Practitioner, Basildon & Thurrock University Hospital, Basildon, UK; Janet Phipps, South Warwickshire NHS Foundation Trust, Warwick, UK; Erini Platsa, St Thomas's Hospital, London, UK; Claire Prince, Queen's Hospital, Burton Upon Trent, UK; Helen Probert, Royal Cornwall Hospital, Truro, UK; Jane Radford, Queen's Hospital, Burton Upon Trent, UK; Lucy Rashley, St Mary's Hospital, Newport - Isle of Wight, UK; Heidi Ribchester, Southport & Ormskirk Hospital, Southport , UK; Katrina Rigby, Royal Preston Hospital, Preston, UK; Lindsay Roughley, Warrington and Halton Hospitals NHS Foundation Trust, Warrington, UK; Elizabeth Simcock, West Cumberland Hospital, Whitehaven, Cumbria, UK; Donna Southam, Basildon & Thurrock

University Hospitals, Basildon, UK; Jennifer Syson, Bradford Teaching Hospitals NHS Trust, Bradford, UK; Yvonne Toomassi, Nottingham City Hospital, Nottingham University Hospitals NHS Trust, Nottingham, UK; Paula Trinham, Heart of England NHS Foundation Trust, Birmingham, UK; Louise Underwood, Chesterfield Royal Hospital, Chesterfield, UK; Eileen Walton, City Hospital, Sunderland, UK; Jane Wardlaw, The Dudley Group of Hospitals, Dudley, UK; Fiona Yelnoorkar, Royal Victoria Infirmary, Newcastle University Teaching Hospitals, Newcastle, UK.

We are also grateful to Paul Piette, Scientific & Medical Affairs Director, Senior Research Fellow, Besins Healthcare Corporate, Brussels, Belgium and to Besins Healthcare for their kind donation of active and placebo drug for use in the study; and to staff of the pharmacy departments and R&D departments of the participating hospitals.

Lastly, we are grateful to the many people who helped in this study but who we have been unable to name, and in particular all the women (and their babies) who participated in OPPTIMUM.
